# Deficiency of *Leishmania* Phosphoglycans Influences the Magnitude but Does Not Affect the Quality of Secondary (Memory) Anti-*Leishmania* Immunity

**DOI:** 10.1371/journal.pone.0066058

**Published:** 2013-06-11

**Authors:** Dong Liu, Ifeoma Okwor, Zhirong Mou, Stephen M. Beverley, Jude E. Uzonna

**Affiliations:** 1 Department of Immunology, University of Manitoba, Winnipeg, MB, Canada; 2 Department of Medical Microbiology, University of Manitoba, Winnipeg, MB, Canada; 3 Department of Molecular Microbiology, Washington University School of Medicine, St Louis, Missouri, United States of America; INRS - Institut Armand Frappier, Canada

## Abstract

Despite inducing very low IFN-γ response and highly attenuated *in vivo*, infection of mice with phosphoglycan (PG) deficient *Leishmania major* (*lpg2-*) induces protection against virulent *L. major* challenge. Here, we show that mice infected with *lpg2- L. major* generate *Leishmania*-specific memory T cells. However, *in vitro* and *in vivo* proliferation, IL-10 and IFN-γ production by *lpg2*- induced memory cells were impaired in comparison to those induced by wild type (WT) parasites. Interestingly, TNF recall response was comparable to WT infected mice. Despite the impaired proliferation and IFN-γ response, *lpg2-* infected mice were protected against virulent *L. major* challenge and their T cells mediated efficient infection-induced immunity. *In vivo* depletion and neutralization studies with mAbs demonstrated that *lpg2- L. major*-induced resistance was strongly dependent on IFN-γ, but independent of TNF and CD8^+^ T cells. Collectively, these data show that the effectiveness of secondary anti-*Leishmania* immunity depends on the quality (and not the magnitude) of IFN-γ response. These observations provide further support for consideration of *lpg2- L. major* as a live-attenuated candidate for leishmanization in humans since it protects strongly against virulent challenge, without inducing pathology in infected animals.

## Introduction

Leishmaniasis is a serious global health problem that affects millions of people worldwide, especially in developing tropical and subtropical countries. According to WHO estimate, about 2 million new cases occur every year and over 12 million people are presently infected [1]. In India, around 60,000 deaths were reported in 1999 due to visceral leishmaniasis, but the actual number is thought to be even much higher [2]. A most recent report estimated that 20,000–40,000 leishmaniasis deaths occur per year and most of these deaths occur in only six countries [3]. Various forms of pentavalent antimonial components are used for treatment of human leishmaniasis, but treatment failures and drug resistances are common [4], [5]. Hence, there is urgent need for new drugs as well as the development of effective human vaccines to prevent the disease. The development of effective vaccine requires an understanding of the factors that regulate secondary protective immunity.

Following recovery from primary natural or experimental infection with *L. major*, a state of immunity develops that is able to rapidly protect healed animals (both humans and mice) against secondary challenge [6]. Such infection-acquired immunity is very durable and is mediated by IFN-γ-producing effector and central memory-like T cells [7]. Infection-acquired immunity is the underlying principle behind leishmanization, which is still practiced in some countries today [8]. However, the significant morbidity associated with the practice has hampered its use as an acceptable vaccination strategy. Moreover, because leishmanization results in a chronic (latent) infection state, concerns have been raised of the possibility to full blown infection in immunocompromised individuals [9].

An increasing number of parasites lines arising through gene replacement methods have been described which show some promise as vaccine candidates in animal studies [10]–[12]. Among these, phosphoglycan (PG) deficient *L. major* (termed *lpg2*−) is of particular interest because it does not induce pathology even in immunocompromised mice [13] and persists indefinitely at levels comparable to WT parasites. Persistence has been associated with maintenance of infection-acquired immunity [14], [15]. Since *lpg2−* mutant parasites persist without causing any disease even in the susceptible mice and protects against virulent challenge [12], these attributes make *lpg2−* mutants a promising live-attenuated *Leishmania* vaccine candidate and have provoked considerable interests in understanding how it persists and interacts with the host immune system. An unanswered important question is whether *lpg2−* parasites could induce secondary (memory) immune response comparable to those of WT parasites. Here, we show that despite significant differences in quantity, the secondary anti-*Leishmania* immunity induced by WT and *lpg2−* parasites are qualitatively similar. These findings further support the consideration of *lpg2−* parasites as live attenuated vaccine candidate against cutaneous leishmaniasis.

## Materials and Methods

### Mice

Female C57BL/6 and BALB/c mice 6 to 8-wk-old were purchased from the Central Animal Care Services (CACS), University of Manitoba. Female B6.PL-Thy1a/CyJ (Thy1.1) mice were purchased from Jackson Lab, Bar Harbor, Maine.

### Ethics Statement

All mice were kept at the University of Manitoba Central Animal Care Services (CACS) facility in accordance to the Canadian Council for Animal Care guidelines. The University of Manitoba Animal Use Ethics Committee approved all studies involving animals, including infection, humane endpoints, euthanasia and collection of samples.

### Parasites

The origin of wild type (WT) and phosphoglycan deficient (*lpg2−) L. major* has been previously described [13], [16]–[18]. Parasites were cultured at 26°C in M199 medium (Hyclone, Logan, UT) supplemented with 10% heat-inactivated FBS, 2 mM L-glutamine, 100 U/ml penicillin, 100 µg/ml streptomycin (Hyclone). For selective growth of *lpg2−* line, Hygromycin B (20 µg/ml) was added to the culture media. While in a previous study we reported the occurrence of *lpg2*− revertants lacking LPG but conferring pathology [19], this was not observed in the studies described here.

### Infection Protocol and Parasite Quantification

Stationary phase promastigotes were washed three times, resuspended in PBS at 10^8^/ml and 50 µl containing 5×10^6^ (for C57BL/6 infections) or 2×10^6^ (for BALB/c infections) parasites was injected into the right or left hind footpad. Lesion sizes were monitored weekly with Vanier calipers and parasite burden was determined by limiting dilution assay [20].

### Generation of Bone Marrow Derived Dendritic Cells (BMDCs) and *in vitro* Infection

Bone marrow cells were isolated from the femur of C57BL/6 mice, seeded in 100×15 mm Petri dishes at 2×10^5^/ml and differentiated using recombinant murine GM-CSF (20 ng/ml, Peprotech, Indianapolis, IN). The culture media were changed twice on day 3 and on day 6, and on day 7, the non-adherent cells were collected and assessed for the expression of CD11c, CD40, CD80, CD86 and MHC class II by flow cytometry. The purity of DCs was between 85–92% (CD11c^+^ cells). BMDCs were incubated with WT parasites for 5 hours at a BMDC to parasite ratio of 1∶10. Thereafter, free parasites were washed away and infected cells were used in subsequent co-culture experiments.

### T cell Purification, 5- (6-) Carboxyfluorescein Diacetate Succinimidyl Ester (CFSE) Labeling, Adoptive Transfer and Co-culture Experiments

T cells were purified from the spleens or dLNs of infected or naive mice by positive selection using CD90.2 coated microbeads and autoMACS (Auburn, CA) according to the manufacturer’s suggested protocols and labeled with CFSE dye as described previously [21]. Ten to 30 million cells were adoptively transferred into naïve congenic (Thy1.1) mice by tail vein injection. Recipient mice were subsequently infected with *L. major* the next day, sacrificed at 5, 14 and 21 days post-challenge to assess proliferation, CD44 and CD62L expression and TNF and IFN-γ production.

### *In vitro* Recall Response, Proliferation and Intracellular Cytokine Staining

At various times after infection, spleen and dLN cells were cultured in complete DMEM medium at 4×10^6^ cells/ml (1 ml/well) in 24-well tissue culture plates and stimulated with soluble *Leishmania* antigen (SLA, 50 µg/ml) as previously described [22]. After 72 hr, the culture supernatants were collected and stored at –20°C until assayed for cytokines by ELISA. For proliferation, CFSE-labeled cells labeled were resuspended at 10^6^/ml, plated onto 96-well round bottom plates and stimulated with SLA or anti-CD3 and anti-CD28 as previously described [23] or co-cultured with *L. major*-infected BMDCs at T cell:BMDC of 100∶1. After 5 days, proliferation was analyzed by flow cytometry. Some cells were used for intracellular cytokine (IL-4, IL-10, TNF and IFN-γ) staining as previously described [24]. Samples were acquired on a FACSCanto II flow cytometer (BD Bioscience, Mississauga, ON, Canada) and analyzed with FlowJo software (TreeStar, Ashland, OR).

### Cytokine ELISAs

The levels of IL-4, IL-10, TNF and IFN-γ in the culture supernatant fluids were determined by sandwich ELISA using antibody pairs and recombinant cytokine standard (BD Biosciences San Jose, CA) according to the manufacturer’s suggested protocols.

### Treatment with anti-IFN-γ, anti-CD8 Monoclonal Antibody (mAb) and TNFR-Ig *in vivo*

Mice infected with WT or *lpg2*− *L. major* were injected intraperitoneally with purified anti- IFN-γ mAb (XMG1.2, 2 mg/mouse) or 30 mg/kg Embrel, a TNFR2-Ig fusion protein that inhibits functional activity of murine TNF *in vivo* [25], [26]. The next day, mice were challenged with *L. major* and antibody or fusion protein treatments were continued weekly for additional 2 weeks. In some experiments, mice were treated with anti-CD8 mAb (TIB210, 1 mg/mouse) 1 day before challenge and for additional 2 weeks (at weekly intervals). This treatment leads to complete and sustained depletion all CD8^+^ cells throughout the treatment period. All mice were sacrificed after 3 weeks to estimate parasite burden.

### Statistical Analysis

Results are shown as the mean ± SEM. A two-tailed Student’s t-test was used to compare means of lesion sizes, parasites burden, and cytokine production from different groups of mice. Significance was considered if p≤0.05 (*, = p≤0.05; **, = p≤0.01 and ***, = p≤0.001).

## Results

### *lpg2^−^ L. major* Parasites Induce Memory T cell Population in the Susceptible and Resistant Mice

**Figure 1:**
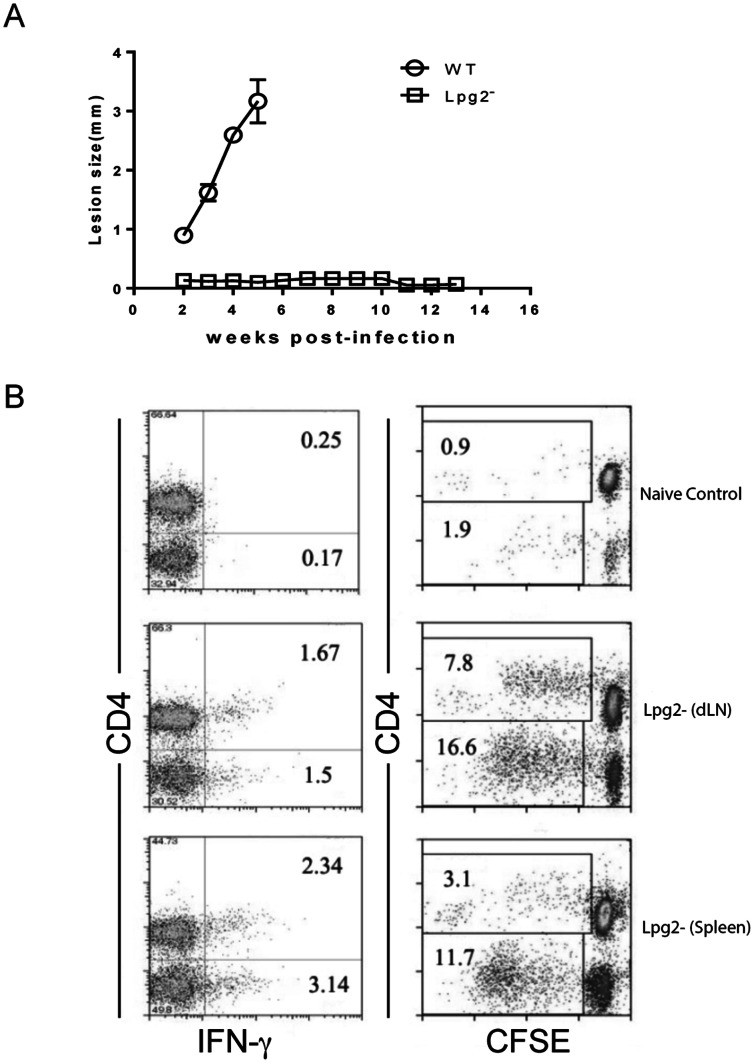
*lpg2− L. major* parasites induce memory T cells in infected BALB/c mice. BALB/c mice were infected with 2 million wild-type (WT) and *lpg2− L. major* stationary phase promastigotes and lesion size was monitored weekly with Vernier calipers (**A**). After 16 wk post-infection, draining lymph node cells and splenocytes from *lpg2−* infected BALB/c mice were labeled with CFSE, restimulated *in vitro* with SLA for 5 days, stained intracellularly for IFN-γ and assessed for proliferation by flow cytometry, the numbers showed on dot plots represent percentages in total T cell population (**B**). Data presented is a representative of 3 independent experiments with similar results.

As previously reported [13], *lpg2^−^ L. major* persists in the footpad of BALB/c mice without causing any lesion for up to 16 weeks post-infection (**Figure 1A** and data not shown). Consistent with our previous observation [12], spleens and draining lymph nodes (dLNs) from 13 wk-infected mice contain low but detectable number of IFN-γ-producing cells (**Figure 1B,** left panel), following short-term (3 days) *in vitro* restimulation with SLA. Furthermore, CD4^+^ T cells from *lpg2-*infected mice (but not those from naïve mice) proliferated strongly in response to SLA stimulation *in vitro*, indicating that parasites-specific memory T cells are maintained even in the absence of cutaneous lesions (**Figure 1B,** right panel).

**Figure 2:**
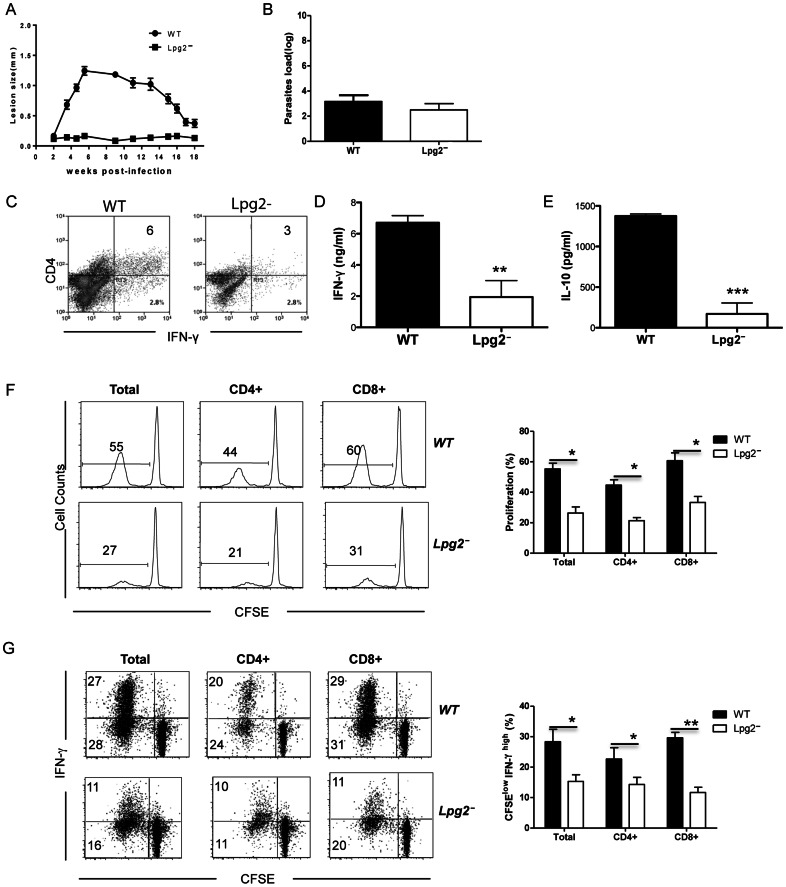
T cells from the spleens of WT and *lpg2−* infected C57BL/6 mice proliferate and produce IFN-γ in response to *L. major*–infected DCs. C57BL/6 mice were infected with WT and *lpg2− L. major* and the kinetics of lesion development and progression was monitored for over 16 weeks (**A**). At 16 weeks after infection, infected mice were sacrificed and parasite burden was determined by limiting dilution (**B**). Spleen cells were restimulated *in vitro* with SLA for 72 hr and the frequency of IFN-γ-producing cells was determined by flow cytometry (**C**). The culture supernatant fluids were assessed for IFN-γ (**D**) and IL-10 (**E**) by ELISA. In some experiments, CFSE-labeled purified T cells purified from infected mice were co-cultured for 5 days with *L. major*–infected BMDCs (T:BMDC = 100∶1), stained for surface expression of CD4 and CD8 and intracellularly for IFN-γ and analyzed by flow cytometry. Shown are the percentages of cells that proliferated i.e. diluted CFSE dye (**F**) and produce IFN-γ (**G**). Data are presented are representative of 2 independent experiments with similar results.

BALB/c mice do not naturally heal WT *L. major* infections, which makes it difficult to compare memory responses following *lpg2*− and WT *L. major* infections in this mouse strain. Therefore, we utilized C57BL/6 mice to investigate and compare the quality of memory response following infection with WT and *lpg2*− *L. major.* As shown in **Figure 2A** and consistent with our previous report [27], *lpg2−*infected C57BL/6 mice did not develop any lesion while mice infected with WT parasites developed lesions that healed by 12 weeks post-infection. Sixteen (16) weeks after infection, both WT and *lpg2−* infected C57BL/6 mice contain comparable numbers of parasites (∼1000) in their footpads (**Figure 2B**). However, the dLNs (data not shown) and spleens of mice infected with *lpg2−* parasites contained significantly less IFN-γ- (**Figure 2C**), IL-4 and IL-10 (**Figure S1A and S1B**) -producing cells and produced less IFN-γ (**Figure 2D**) and IL-10 (**Figure 2E**) than those from WT *L. major*-infected mice following *in vitro* restimulation with SLA.

Because WT and *lpg2− L. major* differentially activate infected dendritic cells [28], we tested whether the impaired IFN-γ response in *lpg2− L. major*-infected mice was related to suboptimal antigen presentation following SLA restimulation. Therefore, we restimulated cells from *lpg2−* infected mice *in vitro* with *L. major*-infected bone marrow-derived dendritic cells (BMDCs) and assessed proliferation and IFN-γ production. Similar to SLA stimulation, proliferation and IFN-γ production by T cells from *lpg2-* infected mice were significantly lower than those from WT-infected mice (**Figure 2F and 2G**). Interestingly, the frequency of TNF-producing T cells from WT and *lpg2*− infected mice following *in vitro* restimulation with infected DCs was comparable (**Figure S2**), suggesting that the difference in IFN-γ recall responses was not related to global impairment or reduction in numbers of *Leishmania*-specific memory cells in *lpg2*- infected mice. Collectively, these results suggest that *lpg2−* parasites are less effective in inducing IFN-γ-producing memory T cell responses than WT parasites.

### Impaired Proliferation and IFN-γ Production by T cells from *lpg2− L. major*-infected Mice are Maintained *in vivo*

**Figure 3:**
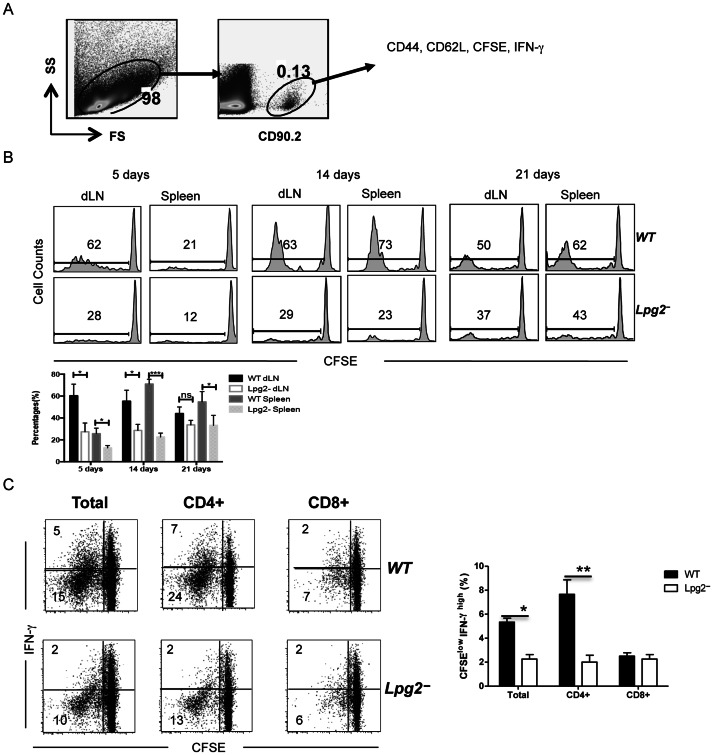
Impaired proliferative response and IFN-γ production by T cells from *lpg2-*infected mice *in vivo*. Naïve Thy1.1 (CD90.1) mice received (by i.v. injection) CFSE-labeled highly purified (>98% pure) T cells from Thy1.2 (CD90.2) mice infected for >16 wk with WT or *lpg2− L. major*. Recipient mice were challenged with 2×10^6^ WT *L. major* the next day and sacrificed at indicated times. Splenocytes or dLNs cells were stimulated with PMA and ionomycin in the presence of Brefeldin A and directly stained with anti-CD3, anti-CD90.2, anti-CD4, anti-CD8, anti-IFN-γ antibodies conjugated with different fluorochromes, gated on CD90.2^+^ donor population (**A**) and analyzed for proliferation (**B**) and IFN-γ production (**C**) by flow cytometry. Data presented are representative of 2 independent experiments (n = 4–5 mice per group) with similar results.

To further determine whether the impaired secondary (proliferation and IFN-γ production) responses observed in T cells from *lpg2− L. major* infected mice are strictly related to T cells and in a more physiological environment, we adoptively transferred equal numbers of CFSE-labeled highly enriched T cells from healed WT and *lpg2− L. major*-infected or naive Thy1.2 (CD90.2^+^) mice into naïve congenic Thy1.1 (CD90.1^+^) recipients and challenged them with WT *L. major*. Challenged mice were sacrificed on days 5, 14 and 21 post-challenge and donor (CD90.2^+^) T cells from the dLNs and spleens were assessed for cell proliferation and IFN-γ production by flow cytometry (**Figure 3A**). At all time points tested, cells from *lpg2-*infected mice were less proliferative (**Figure 3B**) and produced significantly (p<0.05) less IFN-γ (**Figure 3C**) in response to virulent *L. major* challenge than those from WT-infected mice, suggesting that the defect in memory responses observed in *lpg2-*infected mice is strictly related to T cells.

### Quantitative Differences in Memory T cells from WT and *lpg2− L. major*-infected Mice

**Figure 4:**
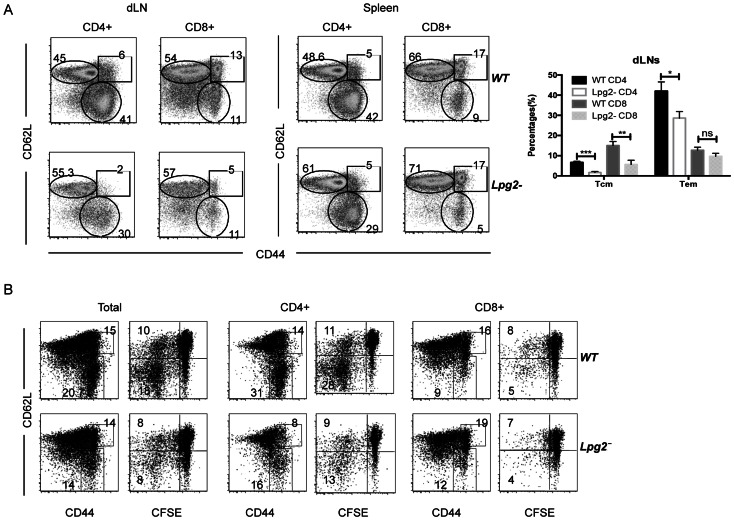
Quantitative differences in memory T cells in WT and *lpg2− L. major*-infected mice. Cells from spleens and dLNs from 20 weeks wide-type and *lpg2–*infected mice were stained with anti-CD3, anti-CD4, anti-CD62L and anti-CD44 antibodies conjugated with different fluorochromes and analyzed by flow cytometry. Expression of CD44 and CD62L on T cell subsets (**A**). Naïve Thy1.1 mice that received CFSE-labeled spleen cells from mice infected with WT or *lpg2- L. major* for >16 wk were challenged 24 hr after cell transfer and sacrificed on day 14 post-challenge. Splenocytes and dLNs cells were stained with anti-CD3, anti-CD90.2, anti-CD4, anti-CD62L and anti-CD44 antibodies conjugated with different fluorochromes and CD90.2^+^ (Thy1.2^+^) cells were analyzed by flow cytometry. The expression of CD44 and CD62L on donor T cell subsets (**B**). Data presented are representative of 2 independent experiments (n = 4–5 mice per group) with similar results.

To directly determine whether there are quantitative differences in numbers of memory T cells in mice infected with WT and *lpg2−* parasites, we assessed the expression of CD62L and CD44 on T (CD3^+^) cells from WT- and *lpg2–*infected mice directly *ex vivo* by flow cytometry. CD44 is a marker of previous T cell activation and hence is expressed by all memory T cells [29] whereas CD62L is a lymph node homing receptor for lymphocytes, which is downregulated upon lymphocyte activation. These markers discriminate between central memory-like T cells (CD44^hi^CD62L^hi^, Tcm) and effector memory-like T cells (CD44^hi^CD62L^lo^, Tem) [24]. Our direct *ex vivo* results show that the percentages of CD4^+^ memory-like T cells (Tcm and/or Tem populations) in the draining lymph nodes of *lpg2−*infected mice were much lower than those from WT-infected mice (**Figure 4A**).

Next, we used the highly sensitive adoptive transfer studies to determine whether there were differences in CD62L expression on proliferating (*Leishmania*-experienced) donor cells from WT and *lpg2− L. major*-infected mice *in vivo*. At day 14 post-challenge, donor CD3^+^ T cells from both groups proliferated and downregulated their CD62L expression (**Figure 4B**), although these events were more pronounced in cells from WT-infected mice. Thus, despite lower proliferative response, *Leishmania*-reactive cells from *lpg2*-infected mice could downregulate their CD62L expression, suggesting that they could potentially home to the site of infection to mediate effector functions.

### Infection with *lpg2^−^ L. major* Protects Against Virulent Challenge Despite Poor DTH Response

**Figure 5:**
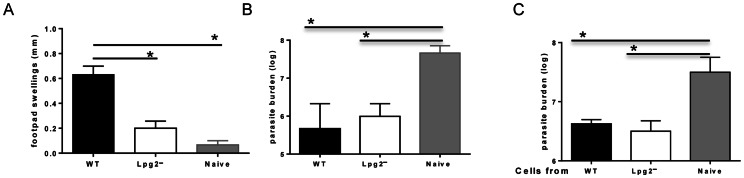
*lpg2− L. major* mediated protection is not associated with a strong DTH response. C57BL/6 mice infected with WT and *lpg2*− *L. major* (>16 wks) were challenge with 5 million WT parasites in their contralateral footpad and delayed DTH response was measured 72 hr post-challenge (**A**)**.** After 3 wk post challenge, mice were sacrificed and parasite burden was determined (**B**)**.** In some experiments, CD3^+^ T cells were purified from spleens of WT or *lpg2-L. major*-infected mice and adoptively transferred into naïve mice that were then challenged with virulent *L. major*. Three weeks after challenge, mice were sacrificed to determine parasite burden (**C**). Data presented are representative of 4 (A and B) and 2 independent experiments (n = 3–5 mice per group) with similar results.

To test whether cells from *lpg2*−infected mice could confer protection to naïve mice following adoptive transfer, we challenged WT and *lpg2-* infected C57BL/6 mice with virulent WT *L. major* parasites and after 72 hr, measured delayed-type hypersensitivity (DTH) response. We found that WT *L. major*-infected mice exhibited strong DTH response whereas *lpg2-*infected mice did not exhibit any significant DTH response following challenge (**Figure 5A**). Interestingly, despite the impaired proliferation, IFN-γ and DTH responses, *lpg2-*infected mice displayed comparable protection to WT-infected mice (**Figure 5B**). Furthermore, adoptive transfer of highly purified T cells from both WT and *lpg2-*infected mice conferred comparable protection to naïve mice against virulent *L. major* challenge (**Figure 5C**). Taken together, these results indicate that despite quantitative differences in recall responses, cells from *lpg2−*infected mice are qualitatively as efficient as those from WT-*L. major*-infected mice in mediating secondary protective immunity.

### Protection in *lpg2−*infected Mice is Dependent on IFN-γ but is Independent of CD8^+^ T cells

**Figure 6:**
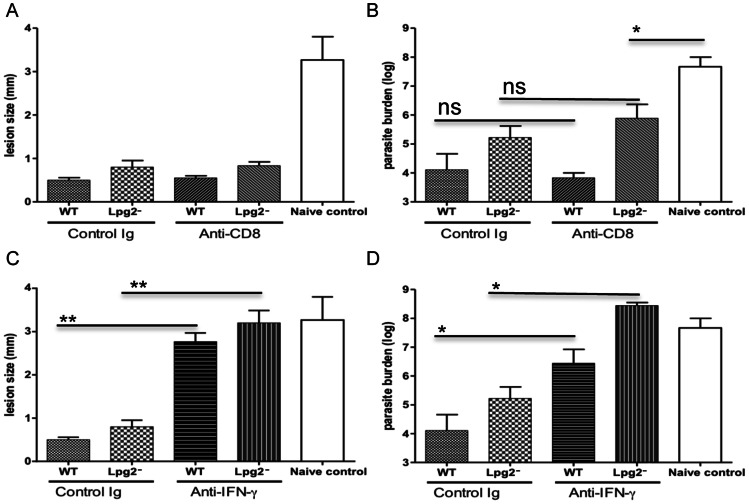
Protection in *lpg2-*infected mice is dependent on IFN-γ but is independent of CD8^+^ T cells. C57BL/6 mice were infected in the right footpad with WT and *lpg2− L. major* and after 16 weeks, challenged with virulent *L. major* in the left footpads. Some mice were treated with anti-IFN-γ or anti-CD8 mAb (1 mg/ml) 24 hr prior to challenge and once weekly for additional 3 weeks. At 3 weeks post-challenge, lesion sizes (**A, C**) were determined and mice were sacrificed and parasite burdens (**B, D**) in the challenged footpads were determined. Data presented are representative of 2–3 independent experiments (n = 3–5 mice per group) with similar results.

We previously showed that infection with *lpg2− L. major* induced a strong primary CD8^+^ T cell proliferation and IFN-γ production [28]. Since CD8^+^ T cells are important in both primary [23], [30] and secondary [31] anti-*Leishmania* immunity, we speculated that secondary resistance in *lpg2*–infected mice might be dependent on CD8^+^ T cells. As shown in **Figure 6A and 6B** and similar to WT infection, depletion of CD8^+^ T cells had no effect on *lpg2-*induced protection, suggesting that CD8^+^ T cells are dispensable for both WT and *lpg2− L. major*-induced immunity. Because *lpg2-*infected mice were protected against secondary *L. major* challenge despite showing significantly impaired IFN-γ recall response *in vitro* and *in vivo*, we investigated whether protection following *lpg2*-infection is independent of IFN-γ. The data in **Figure 6C and 6D** show that IFN-γ is stringently required for *lpg2*-induced protection as neutralization of this cytokine by anti-IFN-γ mAb completely abrogated the protection.

### Protection in *lpg2 L. major-*infected Mice is not due to Intact TNF Production

**Figure 7:**
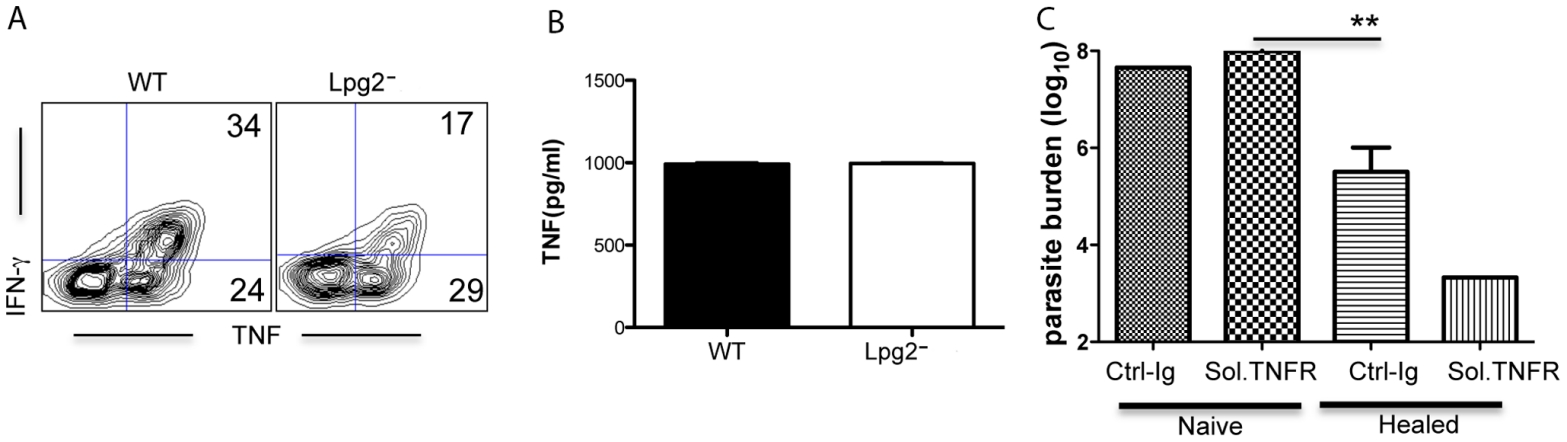
Resistance in *lpg2^−^ L. major*-infected mice is not dependent of TNF. CD3^+^ spleen cells from WT- and *lpg2^−^ L. major*-infected mice were stimulated *in vitro* with infected BMDCs for 24 hr and assessed for IFN-γ and TNF expression by flow cytometer. The culture supernatant fluids were assayed for TNF by ELISA (B). Infected mice were treated with TNFR-Ig (Embrel) 24 hr prior to challenge and further treated once weekly for additional 2 weeks. After 3 weeks mice were sacrificed and parasite burden was determined (**C**)**.** Data presented are representative of 2 independent experiments (n = 3–5 mice per group) with similar results.

In addition to IFN-γ TNF has been shown to play a critical role in resistance to *L. major* [32]. Unlike the impaired IFN-γ expression, the percentage of TNF-expressing CD4^+^ T cells from *lpg2*- infected mice was comparable to those from WT-infected mice (**Figure 7A**) and these cells produced similar amounts of TNF in cultures (**Figure 7B**). Interestingly, the majority (>55%) of cytokine-producing cells in WT-infected mice co-expressed IFN-γ and TNF, suggesting that polyfunctional cells predominate in mice infected with WT but not in those infected with *lpg2* ^−^ parasites. However, treatment of *lpg2*-infected mice with Embrel (soluble TNFR-Ig to block binding of TNF to its cellular receptors) prior to and during secondary *L. major* challenge did not affect *lpg2*-induced resistance (**Figure 7C**), suggesting that *lpg2*-induced resistance is not mediated by TNF.

## Discussion

In this study, we investigated the correlates and possible mechanism of *lpg2-* mediated protection in murine cutaneous leishmaniasis. We demonstrated that *lpg2-*infected mice contain *Leishmania*-reactive (memory) cells that rapidly proliferate and produce effector cytokines (IFN-γ and TNF) in response to *Leishmania* antigen stimulation *in vivo* and *in vitro*. However, *lpg2-* parasites were less effective than WT parasites in inducing and/or maintaining *Leishmania*-specific memory T cells. Nevertheless, memory T cells generated by *lpg2-* parasites were capable of mediating comparable protection against virulent *L. major* challenge, suggesting that *lpg2^–^*induced memory cells are qualitatively and functionally comparable to those induced by WT parasites. Depletion and neutralization studies with mAbs demonstrated that akin to WT parasites, *lpg2- L. major*-mediated resistance was strongly dependent on IFN-γ, but independent of CD8^+^ T cells.

Because of its critical role in resistance to intracellular pathogens, the production of IFN-γ by T cells is widely used as a parameter for assessing vaccine efficiency [33]–[35]. However, our *in vitro* and *in vivo* data demonstrated that cells from *lpg2-*infected mice produced significantly less IFN-γ, yet these mice were strongly protected against virulent *L. major* challenge. These observations suggest that other factors contribute to secondary immunity against virulent *L. major* challenge in *lpg2-* infected mice. Apart from IFN-γ, tumor necrosis factor (TNF) has also been shown to play important role in protective immunity against leishmaniasis [36], [37]. Indeed, the frequency of TNF-producing cells from *lpg2-*infected mice was comparable to those from WT-infected controls (**Figure 7A and 7B**). Neutralization of TNF signaling did not affect resistance of *lpg2* ^−^infected mice to virulent *L. major* challenge, suggesting that TNF does not compensate the defective IFN-γ production in *lpg2-* infected mice. Interestingly, TNF neutralization enhances parasite control in *lpg2- L. major*-infected mice following virulent *L. major* challenge. This apparent paradox may be related to reduced inflammation following TNF neutralization and consequent reduction in macrophage recruitment, which would reduce the number of cells available for parasites to infect.

Although a robust IFN-γ response is important for primary and secondary resistance to *L. major* [38], [39], recent studies suggest that other factors distinct from the magnitude of IFN-γ response might play a more dominant role in regulating the outcome of infection with *L. major.* For example, despite the presence of strong IFN-γ response, *L. major* clone SD (MHOM/SN/74/SD) induces chronic non-healing lesions in C7BL/6 mice that is resolved only after blockade of IL-10 or depletion of CD4^+^CD25^+^ Tregs [40]. In addition, impaired Treg expansion in p110δ deficient mice (and not enhanced IFN-γ response) contributed to the hyper-resistance of these mice to *L. major* infection [41]. These observations suggest that in the absence of Treg activation, low levels of IFN-γ can efficiently activate macrophages leading to effective intracellular parasite killing *in vivo*. However, we did not find any difference in the percentage or absolute numbers of Tregs in both spleens and dLNs of WT and *lpg2*-infected mice (data not shown), suggesting that lower activation of Tregs does not account for the effective primary and/or secondary immunity in *lpg2- L. major* infected mice in the presence of lower IFN-γ response. Interestingly, we found that both primary [12] and secondary (**Figure 2E**) infections with *lpg2^−^ L. major* result in suppressed IL-10 response. It is conceivable that akin to *L. major* infection in p110δ deficient mice [41] and infection with *L. major* clone SD [40], the low IL-10 response could permit low levels of IFN-γ to more efficiently activate macrophages leading to effective parasite killing *in vivo.* Thus, although *lpg2- L. major*-infected mice also produce significantly less IFN-γ, it is conceivable that low levels of IFN-γ may be more efficient at activating macrophages for more efficient parasite control when IL-10 levels are also correspondingly low. In line with this, we found that neutralization of IFN-γ abolished *lpg2*-induced immunity, suggesting that this low level of IFN-γ is nonetheless required for effective parasite control following secondary virulent challenge.

The development of *Leishmania* vaccine is a global public health priority because of the enormous morbidity and mortality associated with the disease. Unfortunately, there is currently no clinical approved vaccine for human cutaneous leishmaniasis [42], [43]. Interestingly, recovery from natural or experimental infection leads to long-lasting protective immunity against re-infection, an observation that formed the basis for leishmanization still practiced in many countries today [44]. However, the significant morbidity associated with this practice has hampered its acceptance as a vaccination strategy. To overcome these problems, a number of live attenuated mutant parasites have been generated [11], [13], [45]–[47]. *lpg2-* parasites have several advantages as a potential live-attenuated vaccine because it does not cause disease in most cases even in the highly immunocompromised (SCID) mice [12]. Although a revertant line of *lpg2*- parasites has been described previously [19], we have yet to detect revertant line in our studies using C57BL/6 mice, suggesting that this may either be a very rare event restricted to the highly susceptible BALB/c mice or due to latent infections or differences in gut microflora resulting from differences in housing environments, a factor that has been shown to affect infection outcome even in animals with same genetic background [48]–[50]. In addition, altered microflora in mice from different vendors (JAX versus The University of Manitoba Central Animal Care Services [originally derived from Charles River, Montreal, Canada]) might also account for the differences from our previous observation regarding the requirement of CpG as adjuvant for generating protective immunity following vaccination with *lpg2-* parasites [27]. Indeed, Dr Beverley’s lab has recently completed a sequence comparison of our strain with theirs (where reversion has been observed) and preliminary analysis show that both strains are genetically the same (i.e. still lack the LPG2 gene), further implicating environmental and/or epigenetic factors as the primary cause of these differences. Furthermore, similar to WT mice, *lpg2-* parasites persist indefinitely in vaccinated mice [13], which eliminates the need for repetitive inoculations. Lastly, *lpg2-* parasites protect vaccinated host against virulent challenge without inducing “nasty” DTH response. We refer DTH as being “nasty” because a huge vaccination-induced swelling (DTH response) on the face following a bite from an infected sandfly would be undesirable.

Overall, our results show that despite poor DTH and IFN-γ recall responses, *lpg2- L. major* parasites induced protective immunity in both BALB/c and C57BL/6 mice is qualitatively comparable to those of WT parasites. We hypothesize that the excellent protection observed in these mice is related to more efficient IFN-γ activity in the presence of low IL-10 response. Our findings lend support for the consideration of *lpg2-* parasites as live-attenuated vaccine or leishmanization candidates against cutaneous leishmaniasis, particularly in parts of the world where leishmanization is still practiced with virulent parasites. This would at least reduce the morbidity associated with using virulent organisms for leishmanization since *lpg2-* parasites do not cause any disease. We are currently examining the pathogenesis of *lpg2-* parasites in non-human primates in order to determine whether infection with this avirulent mutant parasite could also confer protection against virulent challenge.

## Supporting Information

Figure S1 **Impaired IL-4 and IL-10 recall response by spleen cells from** ***lpg2-*** **infected mice.** C57BL/6 mice were infected with WT and *lpg2- L. major* and after 16 weeks, mice were sacrificed, the spleen cells were restimulated *in vitro* with SLA for 72 hr and the frequency of IL-4- (A) and IL-10 (B)-producing cells was determined by flow cytometry.
(TIF)

Figure S2 ***lpg2-*** **infected mice are not impaired in their TNF recall response.** C57BL/6 mice were infected with WT and *lpg2- L. major* and after 16 weeks, mice were sacrificed, the spleen cells were restimulated *in vitro* with infected BMDCs for 72 hr and the frequency of TNF-producing cells was determined by flow cytometry.
(TIF)

## References

[1] Bern C, Maguire JH, Alvar J (2008) Complexities of assessing the disease burden attributable to leishmaniasis. PLoS Negl Trop Dis 2: e313. 18958165 10.1371/journal.pntd.0000313 PMC2569207

[2] Singh N (2006) Drug resistance mechanisms in clinical isolates of Leishmania donovani. Indian J Med Res 123: 411–422. 16778320

[3] Alvar J, Velez ID, Bern C, Herrero M, Desjeux P, et al (2012) Leishmaniasis worldwide and global estimates of its incidence. PLoS One 7: e35671. 22693548 10.1371/journal.pone.0035671 PMC3365071

[4] Chakravarty J, Sundar S (2010) Drug resistance in leishmaniasis. J Glob Infect Dis 2: 167–176. 20606973 10.4103/0974-777X.62887 PMC2889657

[5] Handman E, Kedzierski L, Uboldi AD, Goding JW (2008) Fishing for anti-leishmania drugs: principles and problems. Adv Exp Med Biol 625: 48–60. 18365658 10.1007/978-0-387-77570-8_5

[6] Sacks D, Noben-Trauth N (2002) The immunology of susceptibility and resistance to Leishmania major in mice. Nat Rev Immunol 2: 845–858. 12415308 10.1038/nri933

[7] Scott P, Artis D, Uzonna J, Zaph C (2004) The development of effector and memory T cells in cutaneous leishmaniasis: the implications for vaccine development. Immunol Rev 201: 318–338. 15361250 10.1111/j.0105-2896.2004.00198.x

[8] Khamesipour A, Dowlati Y, Asilian A, Hashemi-Fesharki R, Javadi A, et al (2005) Leishmanization: use of an old method for evaluation of candidate vaccines against leishmaniasis. Vaccine 23: 3642–3648. 15882524 10.1016/j.vaccine.2005.02.015

[9] Puig L, Pradinaud R (2003) Leishmania and HIV co-infection: dermatological manifestations. Ann Trop Med Parasitol 97 Suppl 1107–114. 14678638 10.1179/000349803225002589

[10] Alexander J, Coombs GH, Mottram JC (1998) Leishmania mexicana cysteine proteinase-deficient mutants have attenuated virulence for mice and potentiate a Th1 response. J Immunol 161: 6794–6801. 9862710

[11] Titus RG, Gueiros-Filho FJ, de Freitas LA, Beverley SM (1995) Development of a safe live Leishmania vaccine line by gene replacement. Proc Natl Acad Sci U S A 92: 10267–10271. 7479765 10.1073/pnas.92.22.10267 PMC40777

[12] Uzonna JE, Spath GF, Beverley SM, Scott P (2004) Vaccination with phosphoglycan-deficient Leishmania major protects highly susceptible mice from virulent challenge without inducing a strong Th1 response. J Immunol 172: 3793–3797. 15004184 10.4049/jimmunol.172.6.3793

[13] Spath GF, Lye LF, Segawa H, Sacks DL, Turco SJ, et al (2003) Persistence without pathology in phosphoglycan-deficient Leishmania major. Science 301: 1241–1243. 12947201 10.1126/science.1087499

[14] Uzonna JE, Wei G, Yurkowski D, Bretscher P (2001) Immune elimination of Leishmania major in mice: implications for immune memory, vaccination, and reactivation disease. J Immunol 167: 6967–6974. 11739516 10.4049/jimmunol.167.12.6967

[15] Belkaid Y, Hoffmann KF, Mendez S, Kamhawi S, Udey MC, et al (2001) The role of interleukin (IL)-10 in the persistence of Leishmania major in the skin after healing and the therapeutic potential of anti-IL-10 receptor antibody for sterile cure. J Exp Med 194: 1497–1506. 11714756 10.1084/jem.194.10.1497 PMC2193677

[16] Spath GF, Epstein L, Leader B, Singer SM, Avila HA, et al (2000) Lipophosphoglycan is a virulence factor distinct from related glycoconjugates in the protozoan parasite Leishmania major. Proc Natl Acad Sci U S A 97: 9258–9263. 10908670 10.1073/pnas.160257897 PMC16855

[17] Capul AA, Hickerson S, Barron T, Turco SJ, Beverley SM (2007) Comparisons of mutants lacking the Golgi UDP-Galactose or GDP-Mannose transporters establish that phosphoglycans are important for promastigote but not amastigote virulence in Leishmania major. Infect Immun. 10.1128/IAI.00735-07 PMC1951182 17606605

[18] Capul AA, Barron T, Dobson DE, Turco SJ, Beverley SM (2007) Two functionally divergent UDP-Gal nucleotide sugar transporters participate in phosphoglycan synthesis in Leishmania major. J Biol Chem 282: 14006–14017. 17347153 10.1074/jbc.M610869200 PMC2807729

[19] Spath GF, Lye LF, Segawa H, Turco SJ, Beverley SM (2004) Identification of a compensatory mutant (lpg2-REV) of Leishmania major able to survive as amastigotes within macrophages without LPG2-dependent glycoconjugates and its significance to virulence and immunization strategies. Infect Immun 72: 3622–3627. 15155672 10.1128/IAI.72.6.3622-3627.2004 PMC415719

[20] Titus RG, Marchand M, Boon T, Louis JA (1985) A limiting dilution assay for quantifying Leishmania major in tissues of infected mice. Parasite Immunol 7: 545–555. 3877902 10.1111/j.1365-3024.1985.tb00098.x

[21] Lyons AB, Parish CR (1994) Determination of lymphocyte division by flow cytometry. J Immunol Methods 171: 131–137. 8176234 10.1016/0022-1759(94)90236-4

[22] Scott P, Pearce E, Natovitz P, Sher A (1987) Vaccination against cutaneous leishmaniasis in a murine model. I. Induction of protective immunity with a soluble extract of promastigotes. J Immunol 139: 221–227. 3495599

[23] Uzonna JE, Joyce KL, Scott P (2004) Low dose Leishmania major promotes a transient T helper cell type 2 response that is down-regulated by interferon gamma-producing CD8+ T cells. J Exp Med 199: 1559–1566. 15184505 10.1084/jem.20040172 PMC2211781

[24] Liu D, Uzonna JE (2010) The p110 delta isoform of phosphatidylinositol 3-kinase controls the quality of secondary anti-Leishmania immunity by regulating expansion and effector function of memory T cell subsets. J Immunol 184: 3098–3105. 20154209 10.4049/jimmunol.0903177

[25] Markey KA, Burman AC, Banovic T, Kuns RD, Raffelt NC, et al (2010) Soluble lymphotoxin is an important effector molecule in GVHD and GVL. Blood 115: 122–132. 19789388 10.1182/blood-2009-01-199927

[26] Amante FH, Haque A, Stanley AC, Rivera Fde L, Randall LM, et al (2010) Immune-mediated mechanisms of parasite tissue sequestration during experimental cerebral malaria. J Immunol 185: 3632–3642. 20720206 10.4049/jimmunol.1000944

[27] Kebaier C, Uzonna JE, Beverley SM, Scott P (2006) Immunization with persistent attenuated Delta lpg2 Leishmania major parasites requires adjuvant to provide protective immunity in C57BL/6 mice. Infect Immun 74: 777–780. 16369039 10.1128/IAI.74.1.777-780.2006 PMC1346616

[28] Liu D, Kebaier C, Pakpour N, Capul AA, Beverley SM, et al (2009) Leishmania major phosphoglycans influence the host early immune response by modulating dendritic cell functions. Infect Immun 77: 3272–3283. 19487470 10.1128/IAI.01447-08 PMC2715672

[29] DeGrendele HC, Estess P, Siegelman MH (1997) Requirement for CD44 in activated T cell extravasation into an inflammatory site. Science 278: 672–675. 9381175 10.1126/science.278.5338.672

[30] Belkaid Y, Von Stebut E, Mendez S, Lira R, Caler E, et al (2002) CD8+ T cells are required for primary immunity in C57BL/6 mice following low-dose, intradermal challenge with Leishmania major. J Immunol 168: 3992–4000. 11937556 10.4049/jimmunol.168.8.3992

[31] Muller I, Kropf P, Etges RJ, Louis JA (1993) Gamma interferon response in secondary Leishmania major infection: role of CD8+ T cells. Infect Immun 61: 3730–3738. 8359894 10.1128/iai.61.9.3730-3738.1993 PMC281071

[32] Korner H, McMorran B, Schluter D, Fromm P (2010) The role of TNF in parasitic diseases: still more questions than answers. Int J Parasitol 40: 879–888. 20399786 10.1016/j.ijpara.2010.03.011

[33] Cooper AM, Dalton DK, Stewart TA, Griffin JP, Russell DG, et al (1993) Disseminated tuberculosis in interferon gamma gene-disrupted mice. J Exp Med 178: 2243–2247. 8245795 10.1084/jem.178.6.2243 PMC2191280

[34] Flynn JL, Chan J, Triebold KJ, Dalton DK, Stewart TA, et al (1993) An essential role for interferon gamma in resistance to Mycobacterium tuberculosis infection. J Exp Med 178: 2249–2254. 7504064 10.1084/jem.178.6.2249 PMC2191274

[35] Wang ZE, Reiner SL, Zheng S, Dalton DK, Locksley RM (1994) CD4+ effector cells default to the Th2 pathway in interferon gamma-deficient mice infected with Leishmania major. J Exp Med 179: 1367–1371. 7908325 10.1084/jem.179.4.1367 PMC2191434

[36] Titus RG, Sherry B, Cerami A (1989) Tumor necrosis factor plays a protective role in experimental murine cutaneous leishmaniasis. J Exp Med 170: 2097–2104. 2584936 10.1084/jem.170.6.2097 PMC2189541

[37] Liew FY, Li Y, Yang DM, Severn A, Cox FE (1991) TNF-alpha reverses the disease-exacerbating effect of subcutaneous immunization against murine cutaneous leishmaniasis. Immunology 74: 304–309. 1748478 PMC1384609

[38] Locksley RM, Scott P (1991) Helper T-cell subsets in mouse leishmaniasis: induction, expansion and effector function. Immunol Today 12: A58–61. 1829891 10.1016/S0167-5699(05)80017-9

[39] Reiner SL, Seder RA (1995) T helper cell differentiation in immune response. Curr Opin Immunol 7: 360–366. 7546401 10.1016/0952-7915(95)80111-1

[40] Anderson CF, Mendez S, Sacks DL (2005) Nonhealing infection despite Th1 polarization produced by a strain of Leishmania major in C57BL/6 mice. J Immunol 174: 2934–2941. 15728505 10.4049/jimmunol.174.5.2934

[41] Liu D, Zhang T, Marshall AJ, Okkenhaug K, Vanhaesebroeck B, et al (2009) The p110delta isoform of phosphatidylinositol 3-kinase controls susceptibility to Leishmania major by regulating expansion and tissue homing of regulatory T cells. J Immunol 183: 1921–1933. 19596993 10.4049/jimmunol.0901099

[42] Kedzierski L, Zhu Y, Handman E (2006) Leishmania vaccines: progress and problems. Parasitology 133 Suppl: S87–112 10.1017/S0031182006001831 17274851

[43] Okwor I, Uzonna J (2009) Vaccines and vaccination strategies against human cutaneous leishmaniasis. Hum Vaccin 5: 291–301. 19221514 10.4161/hv.5.5.7607

[44] Greenblatt CL (1988) Cutaneous leishmaniasis: The prospects for a killed vaccine. Parasitol Today 4: 53–54. 15463039 10.1016/0169-4758(88)90067-1

[45] Brodskyn C, Beverley SM, Titus RG (2000) Virulent or avirulent (dhfr-ts-) Leishmania major elicit predominantly a type-1 cytokine response by human cells in vitro. Clin Exp Immunol 119: 299–304. 10632666 10.1046/j.1365-2249.2000.01122.x PMC1905512

[46] Huang C, Turco SJ (1993) Defective galactofuranose addition in lipophosphoglycan biosynthesis in a mutant of Leishmania donovani. J Biol Chem 268: 24060–24066. 8226951

[47] Selvapandiyan A, Duncan R, Debrabant A, Lee N, Sreenivas G, et al (2006) Genetically modified live attenuated parasites as vaccines for leishmaniasis. Indian J Med Res 123: 455–466. 16778323

[48] Singer SM, Nash TE (2000) The role of normal flora in Giardia lamblia infections in mice. J Infect Dis 181: 1510–1512. 10751141 10.1086/315409

[49] Ivanov, II, Atarashi K, Manel N, Brodie EL, Shima T, et al (2009) Induction of intestinal Th17 cells by segmented filamentous bacteria. Cell 139: 485–498. 19836068 10.1016/j.cell.2009.09.033 PMC2796826

[50] Kriegel MA, Sefik E, Hill JA, Wu HJ, Benoist C, et al (2011) Naturally transmitted segmented filamentous bacteria segregate with diabetes protection in nonobese diabetic mice. Proc Natl Acad Sci U S A 108: 11548–11553. 21709219 10.1073/pnas.1108924108 PMC3136249

