# Supplementary figures and images for: Deficiency of Leishmania Phosphoglycans Influences the Magnitude but Does Not Affect the Quality of Secondary (Memory) Anti-Leishmania Immunity
Source: PLoS One. 2013 Jun 11;8(6):e66058. doi: 10.1371/journal.pone.0066058 (PMC3679009; doi:10.1371/journal.pone.0066058)

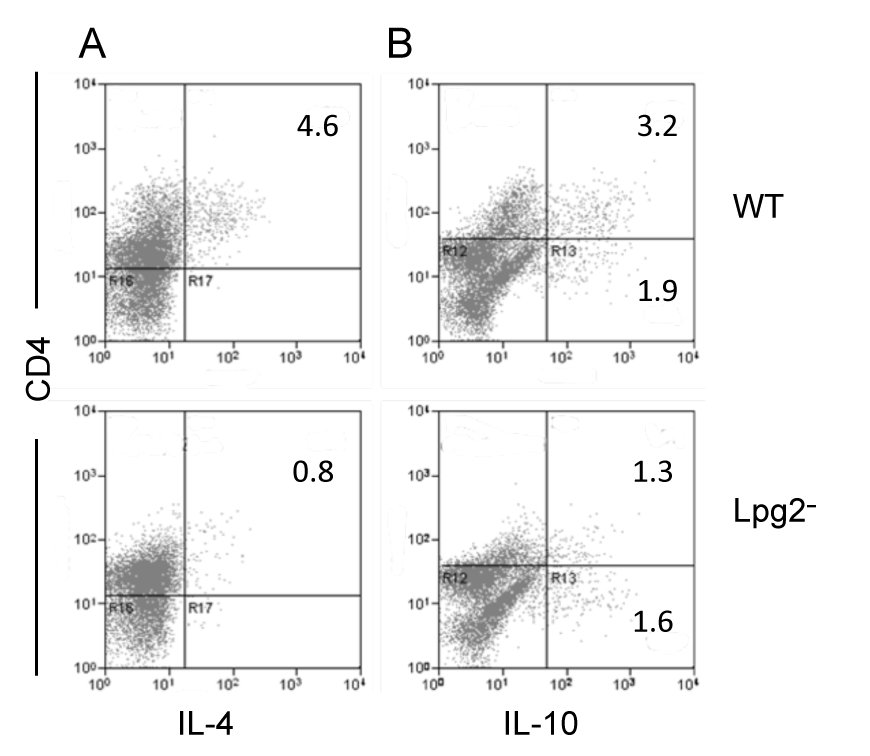

Supplement: Figure S1 — Impaired IL-4 and IL-10 recall response by spleen cells from lpg2- infected mice. C57BL/6 mice were infected with WT and lpg2- L. major and after 16 weeks, mice were sacrificed, the spleen cells were restimulated in vitro with SLA for 72 hr and the frequency of IL-4- (A) and IL-10 (B)-producing cells was determined by flow cytometry. (TIF) [file pone.0066058.s001.tif]

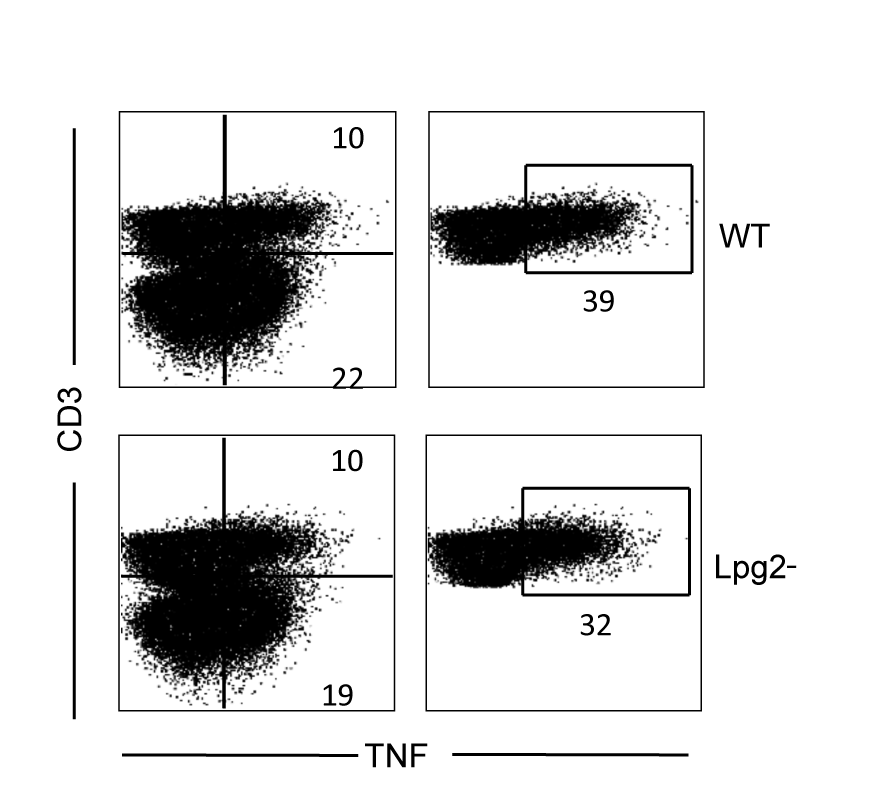

Supplement: Figure S2 — lpg2- infected mice are not impaired in their TNF recall response. C57BL/6 mice were infected with WT and lpg2- L. major and after 16 weeks, mice were sacrificed, the spleen cells were restimulated in vitro with infected BMDCs for 72 hr and the frequency of TNF-producing cells was determined by flow cytometry. (TIF) [file pone.0066058.s002.tif]
